# Relative Low Glycometabolism May Also Occur in Invasive Lung Adenocarcinoma With Visceral Pleural Invasion: Case Report and Comments

**DOI:** 10.1097/MD.0000000000000254

**Published:** 2014-12-05

**Authors:** Chunyang Jiang, Youkui Han, Xiaoli Hu, Bingjun Yang, Tao Tang, Xiangmei Chen, Hui Zhao

**Affiliations:** From the Department of Thoracic Surgery, Tianjin Union Medicine Centre, 190 Jieyuan Road, Hongqiao District, Tianjin 300121 (CJ, YH, BY, HZ); Department of Respiratory Medicine, People's Hospital of Qitaihe City, 37 Shanhu Road, Qitaihe 154600, Heilongjiang (XH); Department of Pathology, Tianjin Union Medicine Centre, 190 Jieyuan Road, Hongqiao District, Tianjin 300121 (TT); and Department of Microbiology & Infectious Disease Center, School of Basic Medical Science, Peking University Health Science Center, 38 Xueyuan Road, Beijing 100191, People's Republic of China (XC)

## Abstract

Lung cancer is the main cause of cancer deaths in the world and positron emission tomography (PET) is considered as the most accurate diagnosis and staging technique for lung cancer. For human cancers, fluorodeoxyglucose (FDG)-PET imaging of most primary and metastatic tumors will show significantly increased glucose uptake because high metabolic activity of cancer cells. But there still have the question of false negative or positive rates in diagnostic accuracy need to be considered.

A 51 year old man was diagnosed a lung tumor in the right middle lobe without enlargement of lymph nodes by computed tomography (CT). The ^18^F-FDG-PET/CT presented 1 slight increased metabolism in the tumor region. After resection of the tumor, postoperative pathological examination confirmed that it was invasive lung adenocarcinoma and with visceral pleural invasion while showed relative low glucose absorption in PET/CT.

A form of invasive lung adenocarcinoma was diagnosized. The tumor tissues were further confirmed by immunohistochemical assessments, which showed that thyroid transcription factor 1 (TTF-1 or NKX2–1) and Cytokeratin 7 (CK7) were all significant positive.

Diagnosis of lung cancer even all other cancers by FDG-PET should be carefully considered the question of accuracy. Our case has added additional literature for us to considering the false-negative of lung cancer diagnosis by ^18^F-FDG-PET/CT.

## INTRODUCTION

Globally, lung cancer was the most commonly diagnosed cancer as well as the leading cause of cancer death in males in recent years. For females, it was the fourth most commonly diagnosed cancer and the second leading cause of cancer death.^1^ Non-small cell lung cancer (NSCLC) accounts for more than 85% of lung cancer cases. Adenocarcinoma is the most common type of lung cancer and accounts for 35% of cases. It is most commonly seen in women and non-smokers. The periphery of the lung especially the upper lobe is the dominant area of localization.^2^ They are further directed to potentially curative treatments including surgical resection, radiotherapy, or combined radiochemotherapy with the intent of curative therapy.^3^

Computed tomography (CT) has been widely used for the initial diagnosis and staging of patients with NSCLC for many years. Positron emission tomography (PET) is a type of nuclear medicine imaging. ^18^F-fluorodeoxyglucose (FDG), which be used in PET imaging scans is a glucose analog that has the similar metabolism pathway with glucose after entering the cell.^4^ Over the last 3 decades, ^18^F-FDG-PET has dramatically increased the accuracy of metabolic mapping of numerous malignancies, with significant impact on the management of cancer patients at different stages of their disease.^5^ In PET/CT systems, 2 type's images are fused and provide combined physiologic information. PET/CT data were found of benefit in various tumors and in different sites and stages of disease course.

Tumors cells including lung cancer cells have a remarkably different metabolism from that of the tissues, which they are derived and have a high metabolic activity and use more glucose.^6^ Integrated PET-CT is a useful method for the determination of invasion of lung cancer to the chest wall and mediastinal invasion. The diagnostic capability of PET/CT in the preoperative staging of NSCLC is superior to that of CT or PET alone.^7^ However, there still have certain false negative or positive rates in diagnostic accuracy need to be further improved. Here, we report 1 patient case with invasive lung adenocarcinoma and visceral pleural invasion but presented relative low glucose absorption.

## CASE REPORT

**FIGURE 1:**
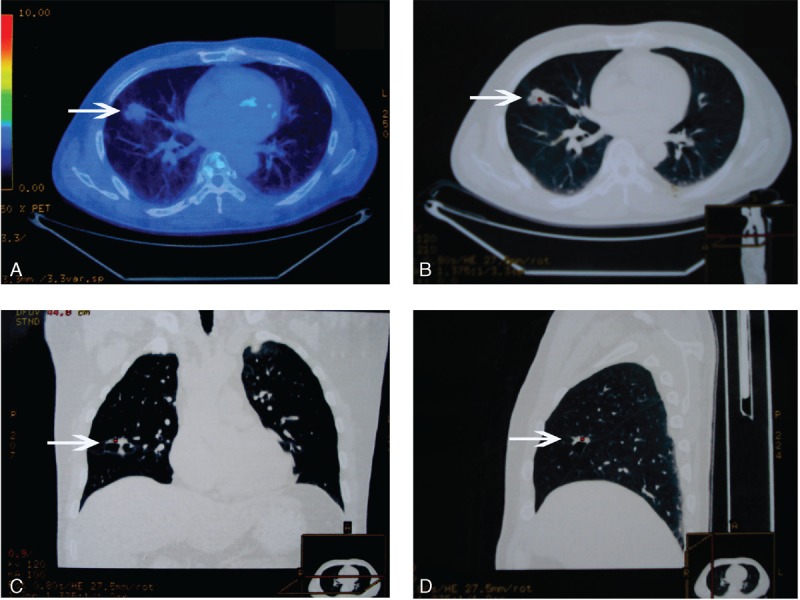
The ^18^F-FDG-PET/CT imaging of the lung lesion. (A) is the fused PET/CT image. (B), (C), and (D) are the CT images which were cross sections, coronal plane, and vertical plane based on the tumor, respectively. The lesion was pointed by white arrows.

The patient was a 51-year-old Chinese Han never-smoker man who, in June 2013, was diagnosed a lung tumor in the right middle lobe with no enlargement of lymph nodes by CT. There was no significant previous medical history was reported. The related examines were finished before operation. No abnormal finding was observed by narrow band imaging (NBI) bronchoscopy. The pulmonary function was normal. The results of Tuberculosis antibody (TB-Ab) and TB-DNA in serum were all negative. Serum tumor markers for lung carcinoma including Carcino-embryonic antigen, Carbohydrate antigen 125, Carbohydrate antigen 72–4, Squmaous cell carcinoma, Cytokeratin 19 fragments, Neuronspecific enolase, and Ferritin were all in normal range. The ^18^F-FDG-PET/CT presented 1 increased density in the right middle lobe with dispersive slight increased metabolism. The standardized uptake value (SUV)_max_ was only about 2.0 and was considered for inflammatory performance (Figure 1).

**FIGURE 2:**
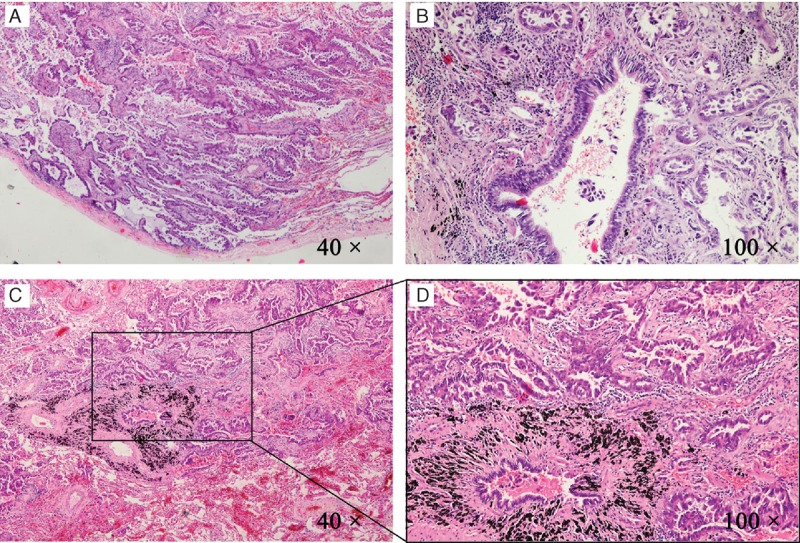
The pathological results of the lung tumor. Lung histopathology changes were observed in light microscope (Nikon Eclipse 80i, Tokyo, Japan) and photos were taken. Shown are the representative images of postoperative pathological results. (A) The tumor tissue and visceral pleura (40×). (B) The tumor tissue around 1 small bronchus (100×). (C) The histopathological tumor tissue arounded by normal lung tissue (40×). Histopathological tumor tissue was present in a further magnification (100×) of the black bordered box in (C) and was shown in (D).

**FIGURE 3:**
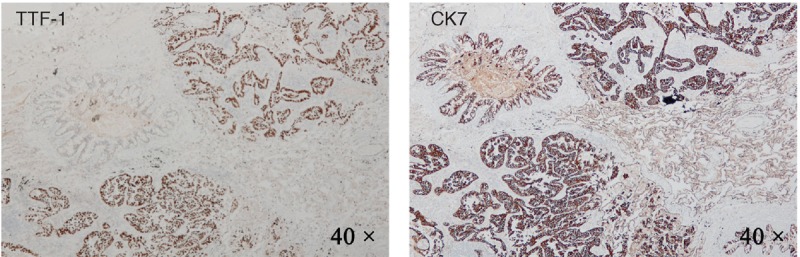
The results of immunohistochemical detection of the tumor tissue. TTF-1 was showed in (A) with magnification of 40×. CK7 was showed in (B) with magnification of 40×.

Subsequently, the lung resection surgery of the right middle lobe was been proceeded by video-assisted thoracoscopic lobectomy. A neoplasm was founded in the lateral section of the right middle lobe, which was hard and globose, 2.0 cm in diameter. Post-operative pathological reports showed that the neoplasm was in the right middle lobe and 4.5 cm from the border of trachea cut, and the volume was 2.5 cm × 1.5 cm × 1.0 cm. Surprisingly, the pathological diagnosis was invasive lung adenocarcinoma and with visceral pleural invasion although had no metastasis in bronchial cut margin and lymph nodes (Figure 2). The results of immunohistochemical assessments showed that Thyroid transcription factor 1 (TTF-1 or NKX2–1) and Cytokeratin 7 (CK7), which are sensitive and specific expressions (positive rate more than 70%) in NSCLC and especially in adenocarcinoma, were all significant positive (Figure 3). Ki-67 positive rate was more than 5%, and part of P53 was positive.

This case report and related experimental protocols were approved by the ethics committee of Tianjin Union Medicine Centre of China. The patient was informed and has consented for the publication of this report.

## DISCUSSION

PET-CT is considered the most accurate staging technique and was strongly suggested should be performed in all patients with NSCLC under consideration for preoperative staging and for surgery.^8,9^ FDG-PET imaging of thousands of oncology patients has unequivocally shown that most primary and metastatic human cancers showing significantly increased glucose uptake.

Tumors with low activity are well-known major causes of false negative findings. ^18^F-FDG-PET results can be false negative in pulmonary carcinoid tumors that are highly differentiated and in bronchioloalveolar carcinoma (BAC) subtype.^10^ Another major cause of false negative findings for malignancy is tumor size. When lesions are smaller than 1 cm, these lesions may not show high FDG uptake in the lungs.^10^ Chronic inflammatory lesions usually do not increase the uptake of FDG.

In this case, the patient had undergone ^18^F-FDG-PET/CT detection and the SUV_max_ of this tumor was relative not significant higher than normal tissue and was considered as chronic inflammatory lesion. However, the post-operative pathological examination, the result was invasive lung adenocarcinoma and with visceral pleural invasion.

Therefore, the PET/CT, although with much accuracy for cancer diagnosis, should not be solely depended absolutely. Abnormal results should be properly interpreted and accurately characterized and could be accomplished only if possible false positive and negative conditions have been aware.

## References

[1] Jemal A Bray F Center MM Global cancer statistics. *CA Cancer J Clin* 2011; 61:69–90. 21296855 10.3322/caac.20107

[2] Aristophanous M Yap JT Killoran JH Four-dimensional positron emission tomography: implications for dose painting of high-uptake regions. *Int J Radiat Oncol* 2011; 80:900–908. 10.1016/j.ijrobp.2010.08.028 20950956

[3] Maurya DK Doi C Kawabata A Therapy with un-engineered naïve rat umbilical cord matrix stem cells markedly inhibits growth of murine lung adenocarcinoma. *BMC Cancer* 2010; 10:590. 21029413 10.1186/1471-2407-10-590 PMC2988749

[4] Tsukamoto N Kojima M Hasegawa M The usefulness of ^18^F-fluorodeoxyglucose positron emission tomography (^18^F-FDG-PET) and a comparison of ^18^F-FDG-PET with 67gallium scintigraphy in the evaluation of lymphoma. *Cancer* 2007; 110:652–659. 17582800 10.1002/cncr.22807

[5] Bar-Shalom R Yefremov N Guralnik L Clinical performance of PET/CT in evaluation of cancer: additional value for diagnostic imaging and patient management. *J Nucl Med* 2003; 44:1200–1209. 12902408

[6] Tennant DA Durán RV Gottlieb E Targeting metabolic transformation for cancer therapy. *Nat Rev Cancer* 2010; 10:267–277. 20300106 10.1038/nrc2817

[7] Fischer B Lassen U Mortensen J Preoperative staging of lung cancer with combined PET-CT. *N Engl J Med* 2009; 361:32–39. 19571281 10.1056/NEJMoa0900043

[8] Ganeshan B Abaleke S Young RCD Texture analysis of non-small cell lung cancer on unenhanced computed tomography: initial evidence for a relationship with tumour glucose metabolism and stage. *Cancer Imaging* 2010; 10:137. 20605762 10.1102/1470-7330.2010.0021 PMC2904029

[9] Fischer BM Mortensen J Hansen H Multimodality approach to mediastinal staging in non-small cell lung cancer. Faults and benefits of PET-CT: a randomised trial. *Thorax* 2011; 66:294–300. 21169287 10.1136/thx.2010.154476

[10] Chang JM Lee HJ Goo JM False positive and false negative FDG-PET scans in various thoracic diseases. *Korean J Radiol* 2006; 7:57–69. 16549957 10.3348/kjr.2006.7.1.57 PMC2667579

